# Vaginal Cuff Closure with a Figure-of-Eight Suture in Total Laparoscopic Hysterectomy: Outcome from 173 Consecutive Cases

**DOI:** 10.3390/medicina62071294

**Published:** 2026-07-04

**Authors:** Nóra Margitai, Olivér Lampé, Adrienne Szilvia Berczi, Rudolf Lampé

**Affiliations:** Department of Obstetrics and Gynecology, Faculty of Medicine, University of Debrecen, 98 Nagyerdei krt., 4032 Debrecen, Hungary; lampe.oliver@med.unideb.hu (O.L.); berczi.adrienn@med.unideb.hu (A.S.B.); lampe.rudolf@med.unideb.hu (R.L.)

**Keywords:** total laparoscopic hysterectomy, operative outcomes, standardization, vaginal cuff closure

## Abstract

*Background and Objectives*: Hysterectomy is one of the most frequently performed gynecological surgical procedures worldwide. It is well established that the laparoscopic approach offers better outcomes for patients compared to abdominal hysterectomy. However, the technique for vaginal cuff closure during laparoscopic hysterectomy (TLH) remains insufficiently standardized in the international literature. Based on our results, we aim to demonstrate that closure of the vaginal cuff using a figure-of-eight suture is a sufficient, reproducible, time-efficient and safe method during total laparoscopic hysterectomy. *Materials and Methods*: Our retrospective observational study analyzed 173 patients who underwent a TLH from January 2016 to December 2021 at the University of Debrecen, Department of Obstetrics and Gynecology. Standardized surgical steps were applied in all cases (ligation of the uterine arteries at their origin, fenestration of the broad ligament above the ureter), and the vaginal cuff was closed laparoscopically with an absorbable suture, incorporating vesicovaginal fascia, vaginal mucosa and uterosacral ligaments. Patient demographics, operative data, and perioperative outcomes were extracted and analyzed. *Results*: TLH was successfully performed in 173 cases, and no conversion to open surgery was necessary. The mean age of the patients was 51.4 (23–86) years, the median body mass index (BMI) was 26.9 (16.9–45) kg/m^2^, and the operative time was 92 (35–240) minutes. The mean uterine weight was 151 (16–440) g. The mean hemoglobin drop was 15.8 (0–44) g/L after the surgery. Regarding complications, ureteral injuries occurred in two cases (1.2%). One patient (0.6%) required relaparotomy due to rectosigmoid perforation. Postoperative complications included vaginal cuff dehiscence in two cases (1.2%), with one patient (0.6%) requiring resuturing, fever in six cases (3.5%), vaginal discharge in four cases (2.3%), and vaginal bleeding in one case (0.6%). Pulmonary embolism occurred in one patient (0.6%). Three patients (1.7%) required blood transfusion based on postoperative blood counts. *Conclusions*: Our results demonstrate an acceptable operative time and a low rate of postoperative complications, suggesting that closing of the vaginal cuff with a figure-of-eight suture is a sufficient and safe technique. This method can be reliably reproduced and incorporated into the standardized steps of total laparoscopic hysterectomy.

## 1. Introduction

Hysterectomy is among the most frequently performed gynecological procedures worldwide [1]. The indications for hysterectomy are diverse, encompassing both benign and malignant uterine pathologies. Approximately 90% of hysterectomies are undertaken for benign conditions, such as abnormal uterine bleeding, leiomyomas, dysmenorrhea, endometriosis, adenomyosis and pelvic organ prolapse [2].

Currently, several surgical techniques are available for performing a hysterectomy. These include open approaches—such as abdominal hysterectomy and abdominal supracervical hysterectomy—as well as minimally invasive methods, including vaginal hysterectomy, laparoscopic-assisted vaginal hysterectomy, laparoscopic hysterectomy, and robot-assisted hysterectomy [3,4,5].

Minimal invasive techniques are associated with a shorter hospital stay, reduced postoperative pain, faster recovery, less blood loss, and lower risk of postoperative infections compared to abdominal approaches [6].

A range of complications may occur following hysterectomy, including bladder injury, ureteral injury, gastrointestinal injury, hemorrhage, postoperative fever, vaginal dehiscence and vaginal vault prolapse [7]. Vaginal cuff dehiscence or evisceration (VCDE) is an infrequent but serious complication of hysterectomy characterized by postoperative separation of the sutured vaginal cuff edges. It is defined as a partial or complete separation of the anterior and posterior vaginal cuff, with or without bowel evisceration, and may result in peritonitis, sepsis, and bowel injury [8].

Identification of specific risk factors for VCD remains challenging. Optimal surgical technique is essential to promote proper cuff healing and reduce the risk of postoperative infection. Preventive measures include avoidance of early sexual intercourse, traumatic vaginal penetration, and excessive strain during the postoperative period. In addition, careful use of electrocautery or other thermal energy, application of delayed-absorbable sutures, and adequate tissue bites may further decrease the risk of dehiscence. Prompt recognition and management are critical to achieving optimal outcomes [9].

Despite the increasing adoption of total laparoscopic hysterectomy (TLH) over abdominal hysterectomy, owing to its numerous advantages, a considerable absence of standardization in operative steps still remains, resulting in different surgical outcomes and challenging quality control [10,11]. A critical step in TLH is the closure of the vaginal cuff, for which no standardized method exists in the international literature. Both institutional practices and individual surgeon preferences contribute to variation in several aspects of cuff closure, including the choice between intracorporeal and transvaginal suturing, the type of suture material used, and the specific suturing technique employed. Variations in closure techniques may significantly affect postoperative outcomes and complication rates [12,13].

Mastering this technique is one of the most essential yet time-consuming components of the learning curve, primarily due to the vaginal wall’s firm structure and limited mobility. Anatomical changes can occur following uterine removal that can lead to retraction of the vaginal vault and displacement of adjacent organs, including the bladder and bowel, into the operative field. These alterations may hinder adequate visualization and access during vaginal cuff closure [14].

The selected method of vaginal cuff closure should aim to preserve vaginal length, provide optimal vault support, and maintain pelvic floor integrity [15].

Evidence from the literature suggests that optimal suturing is achieved when each stitch incorporates approximately 5 mm to 1 cm of tissue on either side and is placed at intervals of about 1 cm. Suturing should commence approximately 5 mm from the lateral angles. Each bite should include the vaginal mucosa, with or without the muscular layer, together with the pubocervical fascia anteriorly and the rectovaginal fascia posteriorly and should also incorporate the posterior peritoneum in the closure [10,16,17].

Apical uterovaginal support may be compromised during TLH if the uterosacral ligaments are not reattached to the vaginal cuff, thereby increasing the risk of subsequent vaginal vault prolapse [18].

Among the various techniques available, the figure-of-eight suture for vaginal cuff closure is noteworthy, though evidence describing its use remains limited in the international literature. This closure technique is performed using absorbable suture material (0/1 Novosyn 90), incorporating the vesicovaginal fascia, the anterior and posterior vaginal walls, and the uterosacral ligaments to provide apical support and minimize the risk of future vault prolapse. This method effectively approximates the free wound edges while promoting favorable wound healing, largely due to the minimal amount of suture material required.

The objective of this study was to analyze total laparoscopic hysterectomy (TLH) procedures performed using standardized surgical steps in which vaginal cuff closure was achieved with a continuous figure-of-eight suture technique and to evaluate whether this method represents a practical, safe, and well-tolerated alternative for surgeons. Specifically, this study aimed to determine whether the figure-of-eight suture is easy to apply, does not increase the rate of vaginal cuff complications, and contributes to reduced operative time.

## 2. Materials and Methods

This retrospective observational study was conducted at the Department of Obstetrics and Gynecology of the University of Debrecen between January 2016 and December 2021. The study was approved by the Institutional Ethics Committee of the University of Debrecen (approval number: DE RKEB/IKEB H.0202-2022) and was conducted in accordance with the Declaration of Helsinki. The requirement for written informed consent was waived due to the retrospective study design and the anonymization of patient data.

In our center, patients were eligible for inclusion if the entire surgical procedure adhered to the standardized steps outlined below and if the vaginal cuff was closed laparoscopically with a figure-of-eight suture. Postoperative complications were monitored and documented up to the 6-week follow-up visit.

Clinical information was obtained from our institution’s electronic surgical database. The study was planned and conducted retrospectively to assess the outcomes of TLH surgeries performed according to our surgical protocol [16]. An initial cohort of 237 TLH cases was identified. Exclusion criteria comprised transvaginal cuff closure, use of suturing techniques other than the figure-eight stitch, any deviation from the protocol and missing data. After applying these criteria, 173 cases remained for the final analysis. Recorded patient characteristics included age, body mass index (BMI), parity, number of previous cesarean and vaginal deliveries, menopause status, comorbidities (such as hypertension, diabetes mellitus, and chronic obstructive pulmonary disease), surgical indication, and history of prior laparotomies and laparoscopies.

Within the surgical dataset, we assessed the following parameters: operative time, uterine weight, duration of vaginal cuff closure using the figure-of-eight suture technique, blood loss, presence of complications (intra- and postoperative) and the type of surgical procedure performed. A group of 4–5 experienced gynecologists performed all procedures and used the same standardized protocol.

The operative time was defined as skin-to-skin suture time excluding anesthesia. The volume of blood loss was calculated from the change in hemoglobin levels between the preoperative measurement and postoperative day 1.

Patients were assessed at 6 weeks postoperatively through medical history and physical examination. All data were obtained from a surgical database, and adverse events were documented according to the Clavien–Dindo classification.

All data were extracted from a prospectively maintained surgical database. Postoperative adverse events were classified according to the Clavien–Dindo classification system and ranged from Grade I to Grade III. No Grade IV or V complications were observed. Grade I complications required neither pharmacological treatment nor surgical intervention and were managed conservatively, such as with analgesics, observation, or intravenous fluid administration, including fever or vaginal discharge. Grade II complications required pharmacological treatment beyond that permitted for Grade I complications or the administration of blood transfusions. Grade III complications were considered severe and required surgical intervention.

Statistical analysis was primarily descriptive. Categorical variables are presented as frequencies and percentages, whereas continuous variables are reported as means with standard deviations (SD) or medians with interquartile ranges (IQR), depending on their distribution. No comparative or inferential statistical tests were performed, as the objective was to assess outcomes within a single cohort without a control group. All analyses were conducted using SPSS version 26.0 (IBM Corp., Armonk, NY, USA).

### Surgical Procedure

All patients underwent a preoperative anesthetic assessment. In line with standard anesthesia guidelines, they were instructed to fast for at least 6 h before surgery. Bowel preparation was not routinely performed. A 2 g dose of Cefazolin was administered to all patients intravenously within 30–60 min before skin incision, according to the institutional perioperative antibiotic prophylaxis protocol.

Low-molecular-weight heparin thromboprophylaxis was initiated 10–12 h after completion of surgery, provided that adequate hemostasis had been confirmed and no contraindication was present.

All procedures were performed under endotracheal anesthesia and with a urinary catheter in place. The catheter was removed 12 h after the surgery. Patients were positioned in lithotomy with a 12–15° Trendelenburg tilt. A reusable metal uterine manipulator consisting of a rigid intrauterine rod and a detachable vaginal fornix cup was used in all cases. This economical, sterilizable, and simple device provides stable uterine positioning and reliable anatomical exposure, facilitating safe colpotomy.

A 10 mm optical trocar with a 30° angled laparoscope was inserted at the umbilicus by direct entry, followed by the placement of three 5 mm trocars under visual guidance. Two lateral (3 cm above the symphysis and 2 cm medial to the anterior superior iliac spine) and one midline trocar were used. Intra-abdominal pressure was set to 15 mmHg. Throughout all procedures, the following devices were used: an ultrasonic cutting device, a bipolar coagulation instrument, blunt forceps, an L-hook electrode, and two needle holders.

The steps of the standard protocol were summarized as follows [19].

1. Right and left pelvic sidewall procedures
  - Coagulation and transection of the round ligament laterally, away from the adnexal and iliac vessels.
  - Dissection of the retroperitoneal space to identify the uterine artery and displace the ureter, followed by ligation of the uterine artery at its origin from the internal iliac artery under direct visualization.
  - Fenestration of the broad ligament above the ureter.
  - Transection of the mesosalpinx and ovarian ligament, or the infundibulopelvic ligament in case of adnexectomy.
2. Mobilization of the urinary bladder
  - Opening the anterior fold of the broad ligament and developing the vesicouterine space with blunt dissection.
3. Dissection of the posterior peritoneum and uterosacral ligament
  - Transection under the guidance of the uterine manipulator with the rotation of the optic.
4. Transection of the cardinal ligament and distal uterine artery
  - Perpendicular dissection under direct visualization of the uterine manipulator.
5. Colpotomy
  - With optical guidance, the procedure began medially on the posterior vaginal wall and proceeded circumferentially around the cervix using monopolar energy in pure cutting mode at 40 W with an L-hook electrode for precise incision.
6. Uterine extraction
  - Removal of the detached uterus by vaginal, intra-abdominal or vaginal morcellation, depending on the uterine size.
  - Intra-abdominal uterine morcellation was performed without the use of an endoscopic retrieval bag and was reserved exclusively for cases with no suspicion of malignancy, based on preoperative assessment and intraoperative findings.
7. Vaginal cuff closure
  - Performing a single intracorporeal figure-of-eight suture laparoscopically, using delayed-absorbable suture material (0/1 Novosyn 90) incorporating the vesicovaginal fascia, the anterior and posterior vaginal walls, and the uterosacral ligaments to provide apical support.

After successful closure of the vaginal cuff, lavage and aspiration were performed, and trocars were removed under direct visualization. Skin closure was performed using an absorbable 4-0 monofilament suture. Postoperative care included administration of analgesics and antiemetics as needed, along with continued thromboprophylaxis for 30 days. Our patients were followed for a six-week follow-up period.

## 3. Results

### 3.1. Demographic Data of Patients

During the study period, 237 patients underwent TLH. A total of 64 patients were excluded from the study because of missing data and exclusion criteria, including transvaginal cuff closure, use of suturing techniques other than the figure-of-eight stitch, deviations from the study protocol, resulting in a final surgical cohort of 173 patients.

Patient demographic characteristics are summarized in Table 1. The mean age of the cohort was 51.4 ± 10.3 years, and the mean body mass index (BMI) was 27.2 ± 5.3 kg/m^2^. A BMI greater than 30 kg/m^2^ was observed in 39 (22.5%) cases. Patients had a parity between 1 and 5. A total of 143 patients (82.6%) had a history of vaginal delivery, and 27 patients had a cesarean section (15.6%) in their medical history. Sixty-two patients (35.8%) were postmenopausal at the time of surgery. A total of 68 patients had a history of laparotomy and/or laparoscopy (39.3%).

### 3.2. Surgical Characteristics, Indications and Outcome Measures

The distribution of surgical indications is presented in Table 2. The most frequent indications for surgery were abnormal uterine bleeding (41.6%), uterine myoma (26.0%), and early-stage endometrial carcinoma (23.7%). In the remaining cases, cervical carcinoma, borderline ovarian tumors, uterine prolapse, and other benign conditions were also identified.

In patients with cervical cancer, laparoscopic hysterectomy was performed in carefully selected cases of low-risk, early-stage disease (FIGO IA1–IA2) following multidisciplinary evaluation and institutional treatment protocols. Surgical management was individualized according to tumor stage, histology, and patient characteristics. The inclusion of patients with cervical cancer in the present study reflects recent changes in the surgical management of early-stage disease. Following the results of the LACC trial, open radical hysterectomy remains the standard treatment for most patients requiring radical surgery. However, less radical approaches have been shown to be safe and effective in carefully selected patients with low-risk disease [20,21].

During data collection, surgical procedures were categorized as total laparoscopic hysterectomy (TLH), total laparoscopic hysterectomy with bilateral salpingo-oophorectomy (TLH-BSO), total laparoscopic hysterectomy with unilateral salpingo-oophorectomy (TLH-USO), total laparoscopic hysterectomy with bilateral salpingectomy (TLH-BS), total laparoscopic hysterectomy with unilateral salpingectomy (TLH-US), and total laparoscopic hysterectomy combined with sentinel lymph node mapping and/or dissection (TLH-LND).

Among the 173 included patients, TLH alone was performed in 2 cases (1.2%), TLH-USO in 6 cases (3.5%), TLH-US in 3 cases (1.7%), TLH-BSO in 101 cases (58.4%), and TLH-BS in 61 cases (35.3%). Within the TLH-BSO group, hysterectomy was combined with sentinel lymph node dissection in 49 cases (48.5%). The mean weight of the excised uterus was 151 ± 78 g. The mean operative time, excluding anesthesia time, was 92 ± 38.1 min. The addition of sentinel lymph node dissection did not result in a significant increase in operative time compared with procedures performed without lymph node dissection. On the first postoperative day, the mean hemoglobin decrease was 15.8 ± 8.7 g/L.

### 3.3. Intraoperative and Postoperative Complications

Table 3 summarizes the intraoperative and postoperative complications according to the Clavien–Dindo classification system.

Among the six patients who developed postoperative fever, no identifiable cause was found in two patients (Clavien–Dindo Grade I), and both recovered with conservative management without the need for pharmacological treatment. The remaining four patients presented with foul-smelling vaginal discharge suggestive of vaginal cuff infection and were successfully treated with antibiotic therapy (Clavien–Dindo Grade II). One of these patients additionally required opening and drainage of the vaginal cuff, which was classified as a Clavien–Dindo Grade III complication. Grade I complications included vaginal bleeding in 1 patient (0.6%), which resolved spontaneously without the need for additional pharmacological or surgical intervention.

Grade II complications included pulmonary embolism in 1 patient (0.6%), vaginal discharge requiring antibiotic therapy in 4 patients (2.3%), and postoperative anemia requiring blood transfusion in 3 patients (1.7%).

Grade III complications were observed in patients requiring surgical intervention. Ureteral injury occurred in 2 cases (1.2%); one injury was recognized intraoperatively and managed laparoscopically by a urologist, whereas the second was diagnosed postoperatively because of urinary retention and subsequently treated. Vaginal cuff dehiscence occurred in 2 patients (1.2%), with 1 patient (0.6%) requiring transvaginal resuturing with absorbable sutures. In the second case, only partial vaginal cuff dehiscence was observed. Because the defect was limited and did not involve complete separation of the vaginal cuff, no surgical intervention was required, and conservative management was sufficient. In addition, 1 patient (0.6%) underwent relaparotomy for rectosigmoid perforation diagnosed on the fourth postoperative day.

## 4. Discussion

Currently, total laparoscopic hysterectomy is increasingly replacing abdominal hysterectomy owing to its advantages of reduced postoperative pain, shorter recovery time, and fewer complications, although laparoscopic suturing of the vaginal cuff remains challenging [7,10]. Mastery of this technique represents one of the most essential yet time-consuming components of the learning curve, primarily due to the firm structure and limited mobility of the vaginal wall. The vaginal cuff is usually created at the level of the vesicovaginal and rectovaginal junctions, producing a relatively straight cuff. Suturing requires a high degree of precision within a confined operative field with limited visibility.

Nevertheless, the literature demonstrates considerable heterogeneity in vaginal cuff closure techniques, with no universally accepted standard, despite the technical complexity involved. Various techniques and suture materials have been developed to address this technical challenge; consequently, barbed sutures have been widely adopted in recent years to simplify the suturing process by eliminating the need for knot tying, thereby reducing operative time and associated complications. Multiple meta-analyses have consistently demonstrated that barbed sutures are both safe and effective, significantly reducing suturing time compared with conventional techniques, while not leading to differences in complication rates or wound healing outcomes. While barbed sutures entail higher material costs, their use also demands specific technical skill, especially when passing the needle through the end of the loop [22,23,24,25,26,27,28]. Smith et al. suggested that reduced operative time may theoretically counterbalance the higher cost of barbed sutures [13].

In a 2018 study by Karacan et al. [27], vaginal cuff closure was performed using barbed V-Loc sutures in 208 patients undergoing total laparoscopic hysterectomy (TLH), whereas conventional figure-of-eight sutures were used in 89 patients. Vaginal cuff dehiscence occurred in three cases in the conventional suture group, while no cases of vaginal cuff separation were observed in the V-Loc suture group. Postoperative vaginal cuff infection or cellulitis was observed in five patients in the standard suture group and in two patients in the barbed suture group. Additionally, the duration of surgery was significantly shorter in the barbed suture group compared with the standard suture group [27].

Planella et al. (2025) [29] compared barbed sutures and conventional sutures for vaginal cuff closure in total laparoscopic and robot-assisted hysterectomies, assessing their effects on operative time, suturing time, blood loss, postoperative complications, surgical site infections, and granulation tissue formation. No significant differences were observed in vaginal cuff dehiscence rates between barbed sutures and conventional sutures. However, in total laparoscopic hysterectomy, the use of barbed sutures was associated with a significant reduction in operative time (by 8.58 min), suturing time (by 4.9 min), and estimated blood loss (by 5.42 mL) [29].

Khoiwal et al. evaluated laparoscopic vaginal cuff closure using unidirectional barbed suture (V-Loc) in 44 patients and a standard polyglactin 910 suture (Vicryl) in 65 patients. The mean vaginal cuff closure time was significantly shorter in the V-Loc group (8.84 min) compared with the Vicryl group (11.66 min). Mean operative time was comparable between the two groups (V-Loc: 109.36 min vs. Vicryl: 108.49 min). Other intraoperative parameters, including blood loss, as well as postoperative outcomes such as pain scores, length of hospital stay, vaginal cuff–related complications (cuff dehiscence, hematoma, or abscess), and dyspareunia, were similar between the groups [22].

Comparing our data with previous studies, closure of the vaginal cuff using a figure-of-eight suture required approximately 8 min, similar to the closure time reported for V-Loc sutures by Khoiwal et al. Despite these comparable cuff closure times, the overall operative time in our series was shorter, averaging 92 min.

It is also important to note that several factors, surgical skills and procedural steps in TLH beyond vaginal cuff closure can influence operative time, which may account for the minimal differences observed in total surgery duration.

Regarding postoperative outcomes, the selected suturing technique may influence the risk of complications, including vaginal cuff dehiscence (VCD). VCD is a rare yet potentially serious complication of hysterectomy with a higher incidence reported following TLH compared with other hysterectomy techniques [30]. In accordance with existing literature, VCD was defined as a partial or complete separation of the vaginal cuff with or without bowel evisceration [17]. Identification of specific risk factors for VCD remains challenging; however, optimal surgical technique is crucial to promote proper healing and reduce postoperative infection. Preventive measures include avoiding early sexual intercourse, traumatic vaginal penetration, and excessive postoperative strain, along with judicious use of electrocautery, delayed-absorbable sutures, and adequate tissue bites.

The influence of suture material and closure technique has been extensively investigated; however, current evidence has not established a clear consensus regarding the optimal method of vaginal cuff closure. Given the complexity of the condition and the interaction of multiple risk factors, the pathogenesis of vaginal cuff dehiscence remains incompletely understood [31].

Prompt recognition and management are essential for optimal outcomes [9,32].

Among our 173 patients, vaginal cuff dehiscence was observed in two cases, with only one requiring resuturing.

In comparison, Karacan et al. reported no vaginal cuff dehiscence among 208 patients who underwent V-Loc suturing [27]. Misirlioglu et al., in a retrospective chart review, analyzed 165 patients who underwent total laparoscopic hysterectomy with vaginal cuff closure using a unidirectional barbed suture technique, without a backward stitch at the distal end. They reported vaginal cuff dehiscence in 2 cases [33].

According to Jeung et al., in a cohort of 248 patients, the risk of vaginal cuff dehiscence was comparable between continuous and figure-of-eight suturing techniques [34].

In the present study, the rates of other postoperative complications-including vaginal bleeding (0.6%), vaginal discharge (1.7%), cuff cellulitis (0%), hematoma (0%)-were comparable to those reported in the studies mentioned above [23,34].

We acknowledge the limitations of our study. Due to the retrospective design, some data were missing, and several patients had to be excluded, which may have affected the study outcomes. Our follow-up was limited to 6 weeks, so we do not have information on long-term outcomes that may have occurred later, such as vaginal cuff prolapse, vaginal cuff integrity, pelvic floor function, and recurrence of symptoms.

## 5. Conclusions

In conclusion, the use of a single figure-of-eight suture for vaginal cuff closure in total laparoscopic hysterectomy appears to be a feasible and safe technique, with low rates of intraoperative and postoperative complications, and can be readily incorporated into routine surgical practice.

Given the design of the present study, no conclusions can be drawn regarding superiority over other established closure techniques, and further comparative studies are required.

This technique may represent a reproducible and easily teachable option for vaginal cuff closure. Although barbed sutures such as V-Loc are widely used, the absence of definitive evidence-based guidelines regarding the optimal closure method highlights the need for continued evaluation of alternative techniques.

## Figures and Tables

**Table 1:** Demographic data of patients.

Patients (*n* = 173)	Mean ± SD or Count
Age (years)	51.4 ± 10.3
BMI (kg/m^2^)	27.2 ± 5.3
BMI > 30 kg/m^2^ (*n*, %)	39 (22.5)
Parity 1–5 (*n*, %)	156 (90.2)
History of vaginal delivery (*n*, %)	143 (82.6)
History of cesarean section (*n*, %)	27 (15.6)
Postmenopausal status (*n*, %)	62 (35.8)

SD = standard deviation, BMI = body mass index.

**Table 2:** Surgical characteristics, indications and outcome measures.

Patients (*n* = 173)	Value (±SD)
Indication: abnormal uterine bleeding (*n*, %)	72
Indication: uterine myoma (*n*, %)	45
Indication: early-stage endometrial carcinoma (*n*, %)	41
Indication: other (*n*, %)	15
Uterine weight (g)	151 (±78)
Total operative time (min)	92 (±38.1)
Hemoglobin drop on postoperative day one (g/L)	15.8 (±8.7)

SD = standard deviation.

**Table 3:** Intraoperative and postoperative complications.

Clavien-Dindo Grade	Complication	*n* (%)	Management
Grade I	Postoperative fever (no identifiable cause)	2 (1.2)	Conservative treatment and observation
Grade I	Vaginal bleeding	1 (0.6)	Spontaneous resolution without intervention
Grade I	Partial vaginal cuff dehiscence	1 (0.6)	Conservative management and follow-up
Grade II	Vaginal discharge	4 (2.3)	Antibiotic therapy
Grade II	Postoperative anemia	3 (1.7)	Blood transfusion
Grade II	Pulmonary embolism	1 (0.6)	Anticoagulant therapy
Grade III	Vaginal cuff infection requiring drainage	1 (0.6)	Opening and drainage of the vaginal cuff
Grade III	Ureteral injury (intraoperative)	1 (0.6)	Laparoscopic repair by urologist under general anesthesia
Grade III	Ureteral injury (postoperative)	1 (0.6)	Surgical/endoscopic management under anesthesia
Grade III	Vaginal cuff dehiscence	1 (0.6)	Transvaginal resuturing with absorbable sutures
Grade III	Rectosigmoid perforation	1 (0.6)	Relaparotomy

## Data Availability

The data presented in this study are available by contacting the corresponding author.
